# Effects of Probiotics on Diarrhea and CD4 Cell Count in People Living With HIV: A Systematic Review and Meta-Analysis

**DOI:** 10.3389/fphar.2021.570520

**Published:** 2021-07-19

**Authors:** Xiao-Li Zhang, Ming-Hui Chen, Shi-Tao Geng, Juehua Yu, Yi-Qun Kuang, Hua-You Luo, Kun-Hua Wang

**Affiliations:** ^1^ NHC Key Laboratory of Drug Addiction Medicine, First Affiliated Hospital of Kunming Medical University, Kunming Medical University, Kunming, China; ^2^ Scientific Research Laboratory Center, First Affiliated Hospital of Kunming Medical University, Kunming, China; ^3^ Yunnan Institute of Digestive Disease, First Affiliated Hospital of Kunming Medical University, Kunming, China

**Keywords:** probiotics, HIV, AIDS, meta-analysis, diarrhea

## Abstract

Gastrointestinal probiotics play an important role in maintaining intestinal bacteria homeostasis. They might benefit people with human immunodeficiency virus/acquired immunodeficiency syndrome (HIV/AIDS), which remains a global health challenge. However, there is a controversy regarding the efficacy of probiotics for the treatment of AIDS. This study systematically reviewed the evidence of the effects of existing probiotic interventions on AIDS and sought to provide information on the role of probiotics in the treatment of HIV/AIDS patients. A meta-analysis of studies identified by screening multiple databases was performed using a fixed-effects model in Review Manager 5.2 software. The meta-analysis showed that probiotics could reduce the incidence of AIDS-related diarrhea (RR = 0.60 (95% CI: 0.44–0.82), *p* = 0.001). The short-term use of probiotics (supplementation duration shorter than 30 days) did not reduce the incidence of diarrhea (RR = 0.76 (95% CI: 0.51–1.14), *p* = 0.19), while the long-term use of probiotics (supplementation duration longer than 30 days) reduced diarrhea (RR = 0.47 (95% CI: 0.29–0.76), *p* = 0.002). Probiotics had no effect on CD4 cell counts in HIV/AIDS patients (MD = 21.24 (95% CI: −12.95–55.39), *p* = 0.22). Our data support that probiotics were associated with an obvious reduction in AIDS-related diarrhea, which indicates the need for additional research on this potential preventive strategy for AIDS.

## Introduction

Acquired immunodeficiency syndrome (AIDS) is a serious chronic condition involving the development of opportunistic infections and other diseases resulting from immune system deficiency and other terrible diseases, leading to death. It is the final phase in the disease progression of human immunodeficiency virus (HIV) infection, which can be sexually transmitted (Roederer et al., 1990). After its emergence, the incidence of AIDS increased annually until it peaked in 1995, after which it declined. The marked decline in the incidence and mortality rates started in 1996 when antiretroviral therapy (ART) was developed and prevention measures were promoted (Suttajit 2007). Although widespread efforts have been made to control the disease and positive results have been realized with ART, AIDS still poses a significant medical challenge (Pavlova-McCalla et al., 2012; Poorolajal et al., 2016). The life-saving pharmacological therapies for HIV/AIDS patients often lead to diarrhea, constipation, and other gastrointestinal conditions (Salminen et al., 2004). Various infections and diarrhea are by far the main causes of death in HIV-positive individuals (Enos et al., 2013). More than half of HIV-positive individuals experience AIDS-related diarrhea, leading to a decline in their quality of life and poor compliance with and tolerance of ART (Suttajit 2007). AIDS-related diarrhea is a major factor contributing to poor adherence to medication. Fortunately, increasing scientific evidence supports the view that probiotic supplements may be therapeutically helpful in the prevention of diarrhea in HIV-positive patients and the reconstitution of a normal gut microbiome (Anukam et al., 2008; Cunningham-Rundles et al., 2000; Irvine et al., 2010; Carter et al., 2016).

Gut-associated lymphoid tissue is a major target site of HIV activity and significantly affects the progression of the disease (Irvine and Hekmat, 2011). The gastrointestinal (GI) tract is usually a site of massive HIV infection, CD4^+^ T-cell depletion, lymphoid tissue fibrosis, aberrant cytokine levels, and enteropathy characterized by enterocyte apoptosis, epithelial tight junction disruption and mucosal inflammation (Anukam et al., 2008; Pinacchio et al., 2018). Bacteria residing in the GI tract can modulate the mucosal immune system, and changes in the mucosal innate immune system can result in the outgrowth of harmful pro-inflammatory bacteria that lead to chronic inflammation in the intestinal mucosa and the periphery (D'Angelo and Costantini, 2017; Liu et al., 2017). Reducing immune activation due to gastroenteritis may thus help slow the progression of the disease. Probiotic microbial populations have important immunoregulatory effects at the level of gut-associated lymphoid tissue (Irvine and Hekmat, 2011), modifying gut bacteria, improving mucosal balance, and regulating allergy-mediated reactions by reducing immunoglobulin E (IgE) antibody production (Heller, 2003). Probiotics have also been shown to decrease the production of interleukin-6 (IL-6) and β2 micro-globulin (Villar-García et al., 2015). Some studies have observed that the elevation of IL-6 is associated with increased morbidity and mortality in HIV-infected patients (Duprez et al., 2012; Kuller et al., 2008). Probiotic bacteria, such as *Bifidobacteria* and *Lactobacillus rhamnosus*, can not only increase the production of short-chain fatty acids and vitamins but also alter the gut epithelium and immunity, improve gut barrier function, help prevent bacterial vaginosis, and reduce the burden of disease (D'Angelo and Costantini, 2017; d'Ettorre et al., 2017; Monachese et al., 2011). Therefore, it is recommended that individuals living with HIV frequently take probiotics as dietary supplements (Lorenc, 2013). However, care should be taken when suggesting probiotic supplementation to patients with HIV/AIDS (Ceccarelli et al., 2019).

The history of probiotics can be traced to the Greeks and Romans, who were the first to recommend the use of cheese and fermented products (Gismondo et al., 1999). The regulatory function of probiotics obtained through dietary supplements may have some important benefits with regard to preventing and treating AIDS-related diarrhea. Here, we conducted a systematic review to summarize the existing scientific evidence on the effects of probiotics on HIV/AIDS patients, identify heterogeneity among the study results, and assess how long should HIV/AIDS patients take probiotics as dietary supplements.

## Methods

### Inclusion Criteria

Included studies met the following criteria: 1) the research supplementation with probiotics in HIV/AIDS patients and patients diagnosed with similar recognized criteria based on laboratory evidence or methods of HIV infection; 2) the ts randomized controlled trials (RCT), clinical cohort studies, pilot study with controls, or other clinical studies; 3) complete data available for HIV/AIDS patients before and after taking probiotics, with the CD4 counts reported as mean ± SDs, and the number of specific incidents of AIDS-related diarrhea (events). When the same groups of patients were reported in multiple articles, only the most recent and complete articles were selected to avoid overlap.

### Exclusion Criteria

The exclusion criteria were as follows: 1) there was no control group in the study; 2) the publication contained the published survey results; 3) the study results were not reported as the mean ± SDs (the CD4 count) and the number of events (AIDS-related diarrhea); 4) the published research data were incomplete and could not be obtained by contacting the corresponding author; 5) the article was a conference paper or abstract; 6) the paper was a duplicate.

### Study Screening

A search was conducted by two independent reviewers using PubMed, Ovid EMBASE, Ovid MEDLINE, Springer, Elsevier Science, the Cochrane Library, Web of Science, and the NIH clinical trial registration system (www.clinicaltrials.gov) from the data of database inception to May 3, 1998. The PICOS framework was used on the search as follows: P, AIDS patients; I, probiotics; C, AIDS patients without probiotic intervention; O, observing the incidence of AIDS-related diarrhea and CD4 cell count in the two groups; S: selecting the study design according to our inclusion criteria. The databases were searched with the terms “probiotics” or “prebiotics”, as well as “Lactobacillus”, “*Bifidobacterium*”, “saccharomyces”, “acquired immunodeficiency disease”, “AIDS”, and “HIV”, according to the Preferred Reporting Items for Systematic Reviews and Meta-Analyses (PRISMA) guidelines. Conference papers, letters to the editor, poster abstracts, reviews, and non-English publications were excluded. Then, the full-text articles were further reviewed for eligibility, and a third reviewer was assigned to settle any disagreements between the reviewers. The references of the identified publications were searched for any additional studies that met the inclusion criteria.

### Data Extraction

Data related to the efficacy of probiotics in HIV/AIDS patients were then reviewed. The data collection form included study, year, country, age, number of research subjects, duration, bacteria species, and dosage (Table 1).

**TABLE 1:** Characteristics of included studies for meta-analysis.

Study, year, country	Subjects ^a^age, number	Duration	Species, dosage
Salminen et al., 2004, Finland	34–54, 17 (8/9)	2 weeks	*Lactobacillus rhamnosus* GG (LGG)LGG, 1–5 × 10^10^ CFU/ml
Anukam et al., 2008, Nigeria	18–44, 24 (12/12)	15 days	*Lactobacillus rhamnosus* GR-1, L.reuteriRC-14, 2.5 × 10^9^ CFU/ml
Irvine et al., 2010, Tanzania	unknown, 166 (85/81)	70 days	*Lactobacillus rhamnosus* GR-1, 1 × 10^9^ CFU/ml
Hummelen et al., 2011a, Canada	≥18, 115 (55/56)	4 weeks	*Lactobacillus rhamnosus* GR-1, 1.23 × 10^9^ CFU/ml
Hemsworth and Reid, 2012, England	38–57, 42 (21/21)	135 days	*Lactobacillus rhamnosus* CAN-1, 1 × 10^9^ CFU/g
González-Hernández et al., 2012, Mexico	22–38, 10 (5/5)	16 weeks	*Lactobacillus rhamnosus* HN001, *Bifidobacterium lactis* Bi-07, 1 × 10^9^ CFU/ml
Schunter et al., 2012, United States	43–55, 27 (13/14)	4 weeks	*Pediococcus pentosaceus* 5–33:3, *Leuconostoc mesenteroides* 32–77:1, *Lactobacillus paracasei* subsp *paracasei* 19, and *Lactobacillus plantarum* 2362
Yang et al., 2014, United States	37–72, 17 (10/7)	12 weeks	*Bacillus coagulans* GBI-30, 1 × 10^9^ CFU/ml
Gautam et al., 2014, India	Unknown, 45 (20/25)	3 months	Probiotic 2.5 × 10^9^ CFU/5 g 10 g for <6 years and 20 g for >6 years (infection duration)
Falasca et al., 2015, Italy	31–53, ^b^30 (30/30)	12 weeks	*Lactobacillus casei shirota*, 6.5 × 10^9^ CFU/bottle (in milk)
Stiksrud et al., 2015, Norway	Unknown, 24 (15/9)	8 weeks	*Lactobacillus rhamnosus* GG (10^8^ CFU/ml), *Bifidobacterium animalis* subsp. lactis B-12 (10^8^ CFU/ml), and *Lactobacillus acidophilus* La-5 (10^7^ CFU/ml) (in milk)
d’Ettorre et al., 2015, Italy	Unknown, ^b^20 (20/20)	48 weeks	*Streptococcus salivarius*, *Bifidobacteria*–represented by *B. breve*, *B. infantis* and *B. longum*, *Lactobacillus acidophilus*, *Lactobacillus plantarum*, *Lactobacillus casei*, *Lactobacillus delbrueckii ssp*. *Bulgaricus*; 204 × 10^9^ CFU/g
Santos et al., 2015, Brazil	≥19, 64 (32/32)	6 months	fructooli-gosaccharides, *Lactobacillus paracasei* (LPC-37), *Lactobacillus rhamnosus* (HN001), *Lactobacillus acidophilus* (NCFM) e *Bifidobacterium lactis* (HN019), 1 × 10^6^–10^9^ CFU/strain
Arnbjerg et al., 2018, Denmark	39–53, ^b^45 (45/45)	8 weeks	*Lactobacillus rhamnosus* GG (LGG)LGG, 6 × 10^10^ CFU/capsule

a years.

b self-control.

### Statistical Analysis

The statistical analysis was performed using Review Manager 5.2 software (Rev Man, Version 5.2, Cochrane Collaboration). Dichotomous and continuous outcome variables are expressed as risk ratios (RRs) and mean differences (MDs), respectively; 95% confidence intervals (CIs) were analyzed as summary statistics. The *χ* ^2^ test was used to evaluate statistical heterogeneity. The *I* ^2^ statistic was used to quantify study heterogeneity, and the extent of inconsistency was assessed by the Cochran Q or the *I* ^2^ statistic. Random- or fixed-effects modeling was applied based on the heterogeneity of outcomes across studies, depending on the *I* ^2^ statistic. An *I* ^2^ of 0%–50% indicates low heterogeneity, 50%–75% moderate heterogeneity, and 75%–100% high heterogeneity. If P(Q) < 0.1 or *I* ^2^ ≥ 50%, a random-effects model was used to calculate the parameters. Conversely, in the absence of heterogeneity, a fixed-effects model was used (Sutton et al., 2000). To identify the origin of the heterogeneity, subgroup analysis and sensitivity analysis were performed. Subgroup analyses were conducted after grouping by probiotic dose, country and region of study, and duration of probiotic supplementation. A sensitivity analysis was performed by excluding studies one by one. A funnel plot was constructed to evaluate their symmetry and visually assess publication bias when the datasets contained at least three studies. A two-tailed *p*-value < 0.05 was considered statistically significant. Two authors (Xiao-Li Zhang and Ming-Hui Chen) used the Newcastle–Ottawa Scale (NOS) to assess study quality. The risk of bias for each included study concerning the efficacy of probiotics in HIV/AIDS patients was also analyzed with the Cochrane tool, which categorizes studies into low, unclear, and high risk of bias in terms of selection bias, detection bias, performance bias, reporting bias, attrition bias, and other sources of bias.

## Results

### Included Studies

**FIGURE 1:**
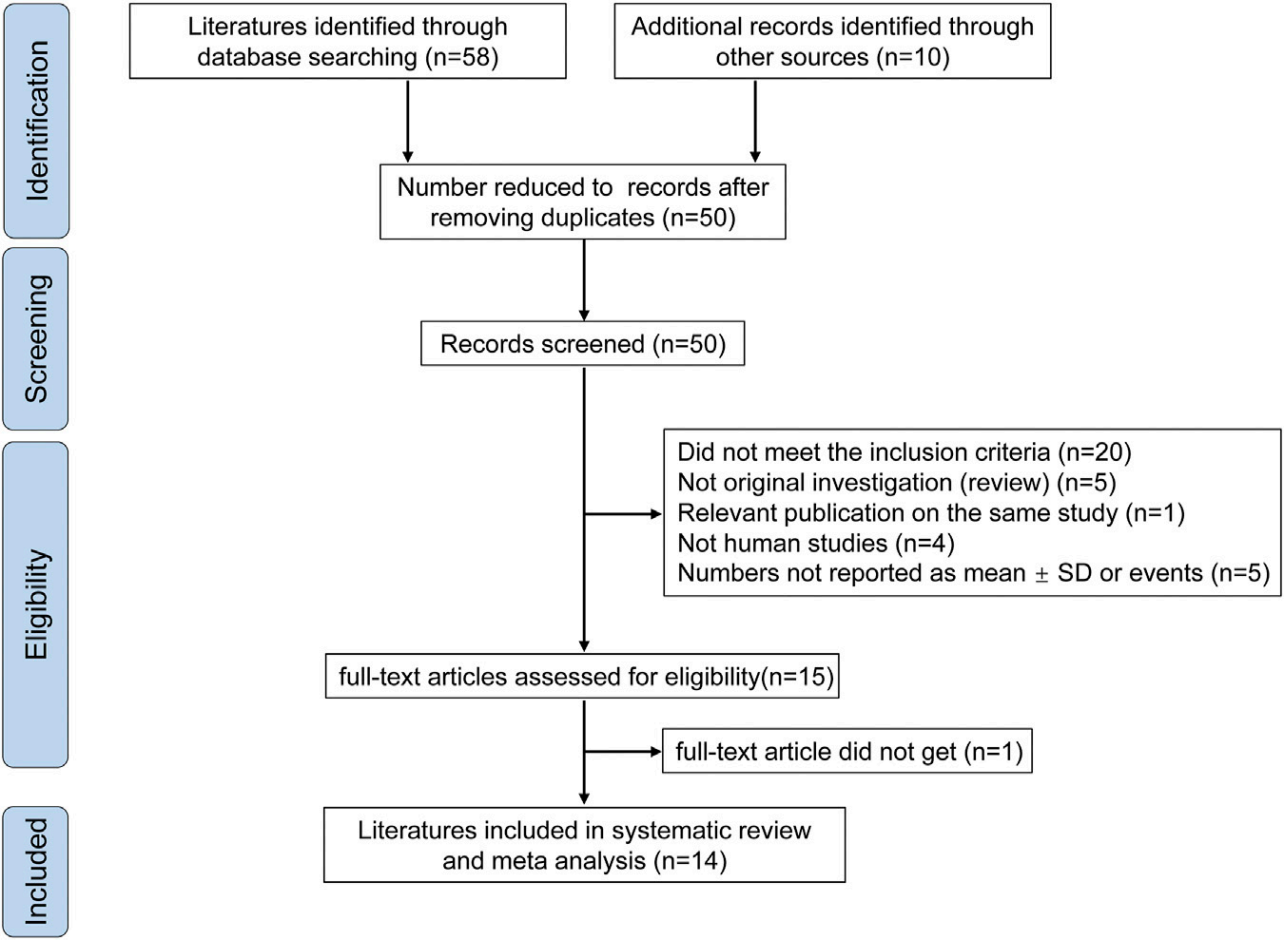
Flowchart for article selection. The flow chart shows a literature search for probiotics on diarrhea and CD4 cell count in people living with HIV.

Sixteen studies were initially identified; however, we excluded two studies (Wolf et al., 1998) (Irvine and Hekmat, 2011) after the sensitivity analysis. Fourteen studies were ultimately included (Anukam et al., 2008; Arnbjerg et al., 2018; d'Ettorre et al., 2015; Falasca et al., 2015; Gautam et al., 2014; González-Hernández et al., 2012; Hemsworth and Reid, 2012; Hummelen et al., 2011a; Irvine et al., 2010; Salminen et al., 2004; Santos et al., 2015; Schunter et al., 2012; Stiksrud et al., 2015; Yang et al., 2014). Ten studies (Anukam et al., 2008; Arnbjerg et al., 2018; d'Ettorre et al., 2015; Falasca et al., 2015; Gautam et al., 2014; González-Hernández et al., 2012; Hemsworth and Reid, 2012; Salminen et al., 2004; Stiksrud et al., 2015; Yang et al., 2014) were included in the analysis of the effect of probiotics on CD4 counts, and six studies (Anukam et al., 2008; Irvine et al., 2010; Hummelen et al., 2011b; González-Hernández et al., 2012; Yang et al., 2014; Santos et al., 2015) were included in the analysis of the effect of probiotics on the incidence of diarrhea. The flow diagram clearly shows the process followed in selecting the studies for this systematic review and meta-analysis (Figure 1). In total, 68 publications were reviewed. The characteristics of the included studies are shown in Table 1. No differences were found in the baseline characteristics of patients in the two groups in the selected studies.

### Quality Assessment

**FIGURE 2:**
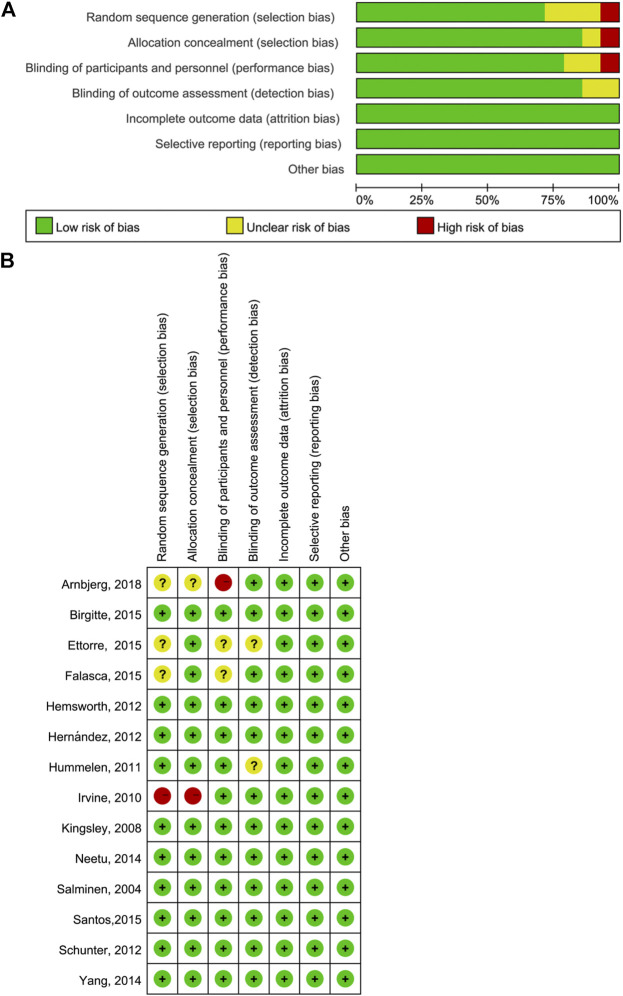
Quality assessment of included literature. **(A)** Bar chart comparing the percent risk of bias for each study included **(B)** Risk of bias for each RCT included. The plus signs indicate a low risk of bias, the subtraction signs represent a high risk of bias, and the question marks denote the unclear risk of bias.

The risk of bias across all studies in the efficacy analysis in each included study is shown in Figure 2. The quality assessment showed that the highest overall risk of bias was in relation to performance and detection.

In assessing the relationship between the CD4 count and probiotics, CD4 counts after probiotics have been taken for a period of time and CD4 counts in patients who did not take probiotics were compared. Initially, 11 studies were evaluated, but the inclusion of one study (Wolf et al., 1998) resulted in a very high *I* ^2^ (48%; *p* = 0.04). After the sensitivity analysis, we excluded this study. The unit of the CD4 count (cells/L) was different from that used in the rest of the studies (cells/μL), and the study was older (1998) than the other studies (2004–2018).

Six studies were included to assess the effect of probiotics on reducing the incidence of AIDS-related diarrhea. Initially, we found seven studies, but one was a retrospective study. The inclusion of that study led to high heterogeneity (*I* ^2^ = 35% and *p* = 0.16), and there is a risk of recall bias in retrospective studies. Therefore, we excluded that study from the analysis.

The probiotic doses in the included studies ranged from 10^9^ to 10^10^ colony forming units (CFU), and the administration duration varied from 15 to 296 days. To ensure the comparability of the studies, short-term use (less than 30 days) and long-term use (more than 30 days) were evaluated separately. All included studies reported baseline data for every group, which were not significantly different.

### Efficacy of Probiotics

**FIGURE 3:**
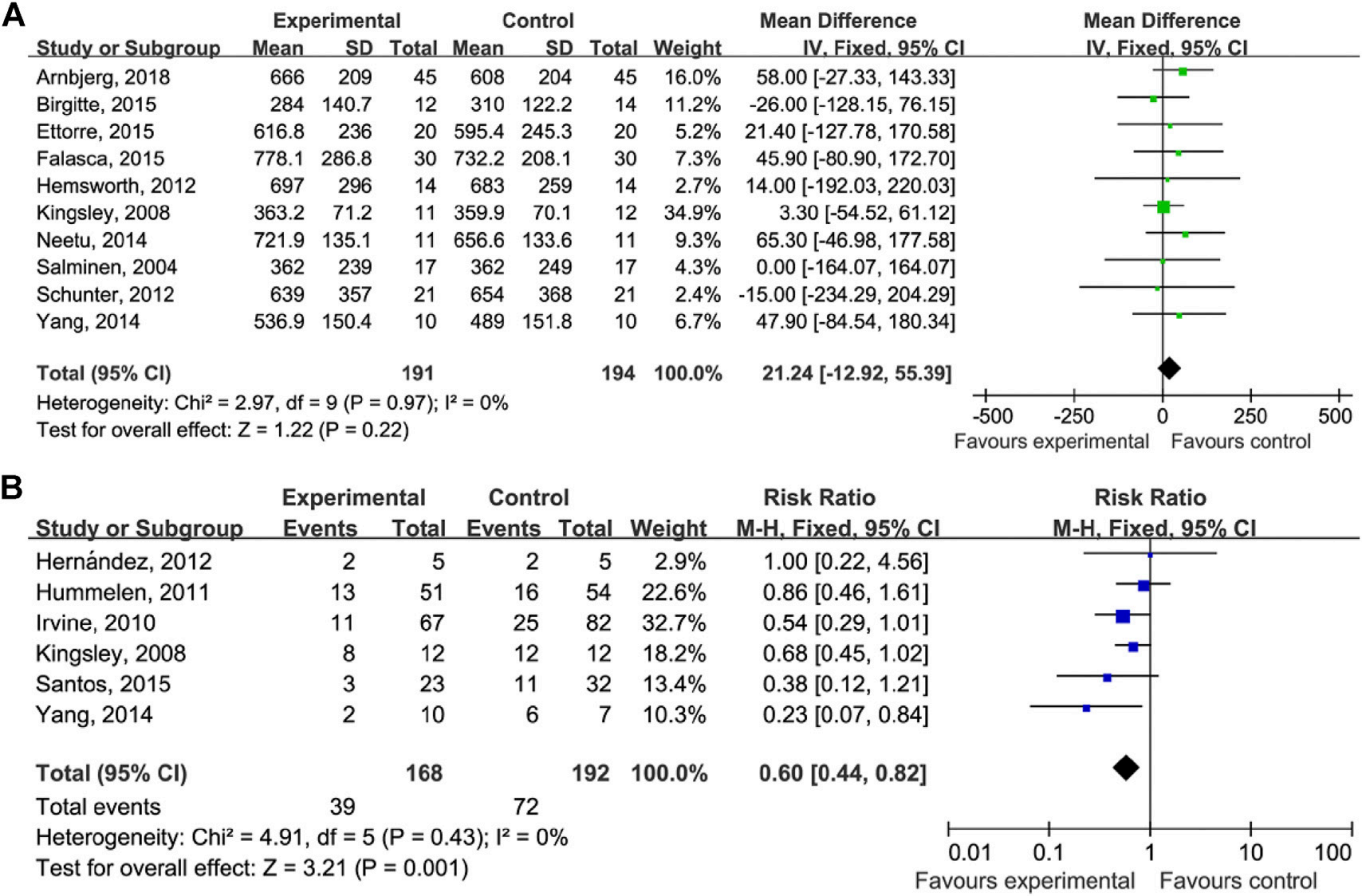
Probiotics on CD4 count and AIDS-related diarrhea. **(A)** Estimates for probiotics associated with CD4 counts in the meta-analysis. **(B)** Estimates for probiotics associated with AIDS-related diarrhea in the meta-analysis.

As shown in Figure 3, the comparison of the CD4 counts between those who did and did not take probiotics showed the following results: MD = 21.24 (95% CI: −12.95–55.39), *p* = 0.22 (Figure 3A), and RR = 0.60 (95% CI: 0.44–0.82), *p* = 0.001 (Figure 3B).

**FIGURE 4:**
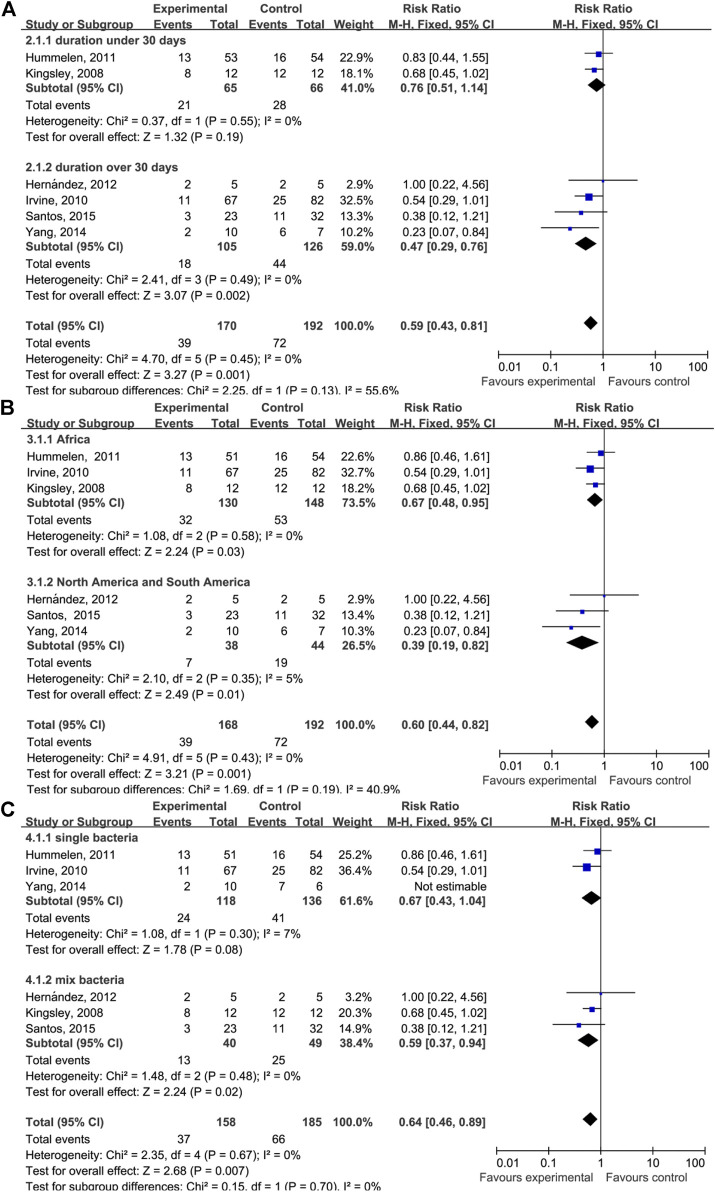
Forest plot analyses of different factors. **(A)** Forest plots for the dosing time of taking probiotics. **(B)** Forest plots for different geographical factors of study subjects. **(C)** Forest plots for single or mixed probiotics of study subjects.

A subgroup analysis was performed to investigate the effect of the duration of probiotics supplementation on the relationship between taking probiotics and the incidence of AIDS-related diarrhea (Figure 4A). The duration of probiotics supplementation in four of the studies was longer than 30 days (RR = 0.47 (95% CI: 0.29–0.76), *p* = 0.002) and the durations in two studies were 15 and 28 days (RR = 0.76 (95% CI: 0.51–1.14), *p* = 0.19). Another subgroup analysis was performed to investigate whether the geographical location and bacterial species affect AIDS-related diarrhea (Figure 4B). We found that studies performed in South or North American countries have reported a decrease in AIDS-related diarrhea events after probiotic supplementation (RR = 0.67 (95% CI: 0.48–0.95), *p* = 0.03). Similarly, studies conducted in Africa have reported a decrease in AIDS-related diarrhea events (RR = 0.39 (95% CI: 0.19–0.82), *p* = 0.01).

Sensitivity analyses were performed to test the stability of the results. We sequentially removed one study at a time and found that doing so did not change the direction of the effect size or the overall heterogeneity, suggesting that the results of this systematic review and meta-analysis were stable.

### Publication Bias

**FIGURE 5:**
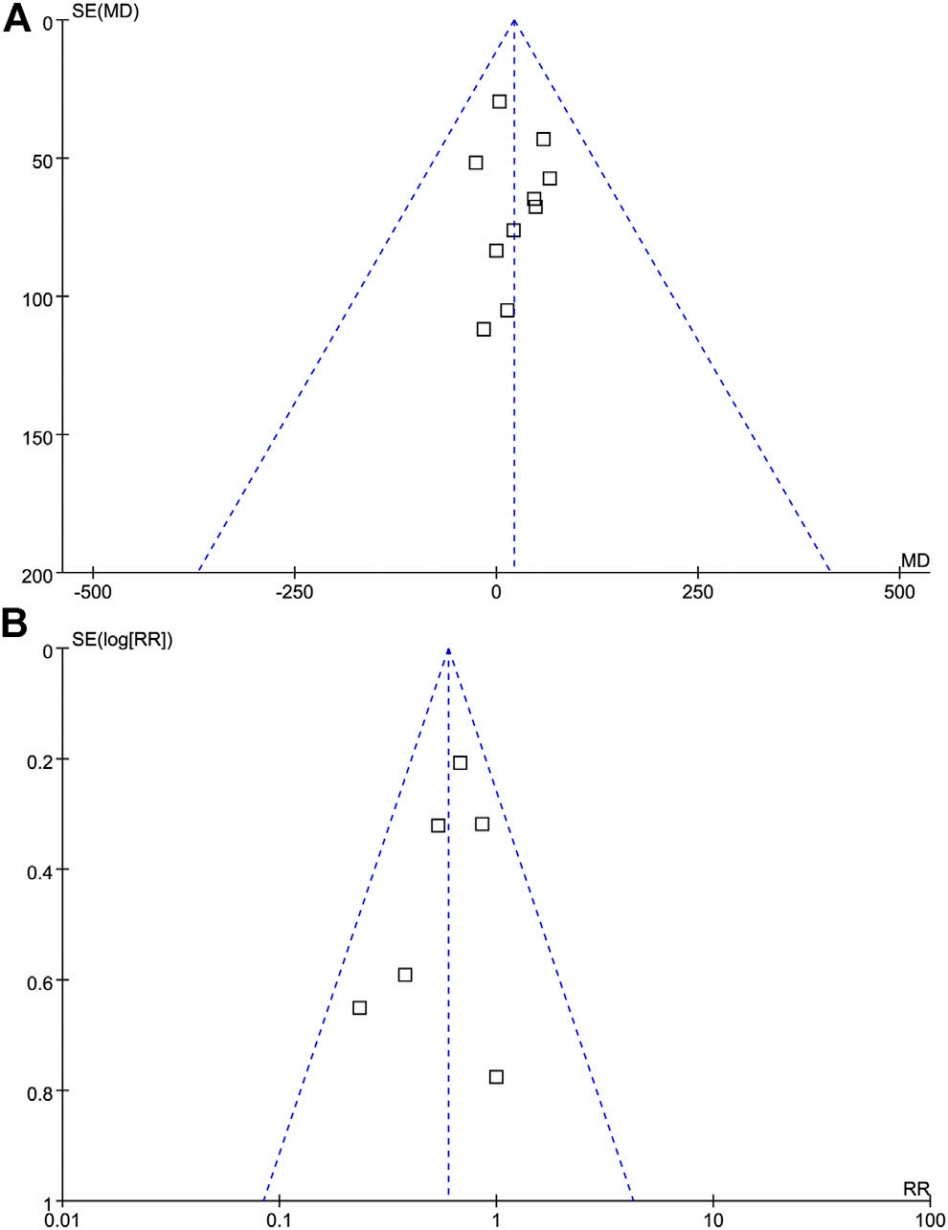
Funnel plot analysis. Funnel Plot detailing publication bias in the studies reporting the impact of probiotics on CD4 cell count **(A)**, and probiotics on the incidence of AIDS-related diarrhea **(B)**. There is no evidence of publication bias.

Publication bias was qualitatively assessed with a funnel plot. As shown in Figure 5, the funnel plot was partially symmetrical but showed no evidence of asymmetry, and therefore, there was no evidence of publication bias among these studies.

## Discussion

The global AIDS epidemic has had severe consequences for humanity. Some HIV/AIDS patients have been treated with ART; however, their quality of life has been seriously affected by acute diarrhea (Suttajit 2007). The treatment of HIV/AIDS can lead to an imbalance in the intestinal bacteria, with a reduction in the populations of some beneficial bacteria, e.g., *Lactobacilli* and *Bifidobacteria* (Vujkovic-Cvijin et al., 2013). Hence, some scientists have recommended that supplementation with some beneficial bacterial species could contribute to the recovery of an adequate mucosal immune response and, to a certain extent, enhance the antiviral defenses. In fact, some studies have supported that probiotic supplementation improved the immune response and quality of living among HIV/AIDS patients (Suttajit 2007). Similarly, our findings suggested that HIV/AIDS patients may benefit from supplemental probiotics, and the meta-analysis showed that probiotics could reduce the incidence of AIDS-related diarrhea (RR = 0.60 (95% CI: 0.44–0.82), *p* = 0.001). Nevertheless, the results of our study make it clear that probiotic supplementation does not increase the CD4 cell count (MD = 21.24 (95% CI: −12.95–55.39), *p* = 0.22).

Functional foods contain probiotics and prebiotics that enhance health-promoting intestinal bacteria in humans. Increasing scientific evidence has suggested that healthy gut bacteria protect against gastrointestinal dysfunction, including gastrointestinal infections, inflammatory bowel diseases, and even cancer (Sharma and Devi 2014). A study conducted by De Simone (De Simone et al., 1992) has evaluated the potential clinical effect of a probiotic containing *Bifidobacterium bifidum* and *Lactobacillus acidophilus* on local and systemic immune responsiveness in elderly individuals. *Bifidobacterium bifidum* and *Lactobacillus acidophilus* treatment can support the integrity and functionality of the mucosal surfaces, improving the immune response and increasing the proliferation of white blood cells (De Simone et al., 1992). These potential impacts were also supported by other studies, such as that by Klatt et al*.*, although that study also showed that treatment with probiotics did not reduce the plasma viral loads or increase the CD4^+^ T-cell count in the blood of SIV-infected animals (Klatt et al., 2013). However, they found that probiotic supplementation in conjunction with ART enhanced the gastrointestinal immune function and promoted the reconstitution of colonic CD4^+^ T-cells. The underlying mechanism could involve an increase in the expression of APC, improving function, and a reduction in the inflammation that causes fibrosis. Therefore, Klatt et al*.* (Klatt et al., 2013) have suggested that probiotics/prebiotics treatment may be an effective supplemental treatment that can be administered in conjunction with ART in HIV-infected individuals to alleviate the residual GI inflammation and damage, thereby potentially having beneficial effects on morbidity and mortality (Klatt et al., 2013). Trois et al. have reached the same conclusion in children (Trois et al., 2008). They investigated 77 HIV-infected children (2–12 years old) and divided the subjects into two groups. In one group, 38 children received probiotics (*Bifidobacterium bifidum* and *Streptococcus thermophilus*) while taking antiviral drugs. In another group, 39 children received a standard formula (control group) for 2 months. The study indicated that probiotics might have immunostimulatory properties and could be beneficial when treating HIV-infected individuals. Other ART regimens were investigated in that study (Trois et al., 2008). It is worth noting that probiotic supplements are safe and effective in HIV-infected individuals, leading to modest reductions in clinical GI symptoms even without any ART treatment (González-Hernández et al., 2012; Hummelen et al., 2011a; Irvine and Hekmat, 2011). In contrast, some studies did not find any effect of probiotics in HIV/AIDS patients and did not observe the preservation of immunologic function during one-month follow-up (Athiyyah et al., 2019; Hummelen et al., 2011b; Roederer et al., 1990). Thus, we conducted the meta-analysis in an attempt to statistically resolve the dispute, as the pooled estimate can indicate whether probiotics have an effect. The superiority of this method is that the aggregation of the data results in a high level of statistical power, yielding robust scientific evidence. We draw the solid conclusion that probiotics can reduce the incidence of AIDS-related diarrhea in HIV/AIDS patients.

The subgroup analysis of probiotic supplementation duration has indicated that, in HIV/AIDS patients who take probiotics for longer than 30 days, the incidence of AIDS-related diarrhea is effectively reduced. No effect was observed among people who took probiotics for fewer than 30 days, indicating that the effects of probiotics on the incidence of AIDS-related diarrhea differ depending on the duration of supplementation. However, because there were only two studies with subjects who took probiotics for fewer than 30 days, this conclusion should be interpreted with caution. Intriguingly, we found that among HIV/AIDS patients who used probiotics for less than 30 days, the type of probiotic was *Lactobacillus rhamnosus GR-1*. To determine whether the use of different probiotics affected the result, subgroups were stratified by geographic location and bacterial species. We found that *Lactobacillus rhamnosus GR-1* was effective in reducing the incidence of diarrhea. It is clear that the duration of probiotic supplementation rather than the probiotic type or geographical location affected the results.

A thorough search of the literature was performed in this systematic review and meta-analysis to reduce publication bias, and each step was executed by two separate researchers to ensure robust results. However, this study had some limitations. First, the dose (from 10^9^ CFU to 10^10^ CFU) and treatment mode (single or mixed bacteria, pharmaceutical dosage, method of administration) of the probiotics investigated in the included studies were different, and other confounding factors such as diet and ART schedule could also have an impact on the study results. In addition, the included studies were from different regions; thus, different genetic backgrounds and exposures to different microbes might have affected the patients’ responses to the same probiotics. Finally, some of the included studies had small sample sizes, reducing the statistical power, which might have affected the reliability and validity of the conclusions.

## Conclusion

This study supports the therapeutic potential of probiotics with regard to reducing the incidence of diarrhea after an administration of one-dose (1 × 10^9^ CFU/ml or 1 × 10^9^ CFU/g) probiotics over 30 days in HIV/AIDS patients. It is noteworthy that this effect strongly depends on the duration of supplementation; we found that it takes longer than 30 days to observe the effects of probiotics. There appears to be no effect of probiotic supplementation for fewer than 30 days on the incidence of AIDS-related diarrhea. More scientific evidence from high-quality studies with larger sample sizes is needed to determine whether probiotics can reduce the incidence of diarrhea in HIV/AIDS patients. In addition, we need stronger evidence to support the safety of probiotics and determine the mechanism by which probiotics reduce the incidence of AIDS-related diarrhea.

## Data Availability

The original contributions presented in the study are included in the article/supplementary material; further inquiries can be directed to the corresponding authors.

## References

[B1] Anukam K. C. Osazuwa E. O. Osadolor H. B. Bruce A. W. Reid G. (2008). Yogurt Containing Probiotic Lactobacillus Rhamnosus GR-1 and L. Reuteri RC-14 Helps Resolve Moderate Diarrhea and Increases CD4 Count in HIV/AIDS Patients. J. Clin. Gastroenterol. 42, 239–243. 10.1097/mcg.0b013e31802c7465 18223503

[B2] Arnbjerg C. J. Vestad B. Hov J. R. Pedersen K. K. Jespersen S. Johannesen H. H. (2018). Effect of Lactobacillus Rhamnosus GG Supplementation on Intestinal Inflammation Assessed by PET/MRI Scans and Gut Microbiota Composition in HIV-Infected Individuals. J. Acquir Immune Defic Syndr. 78, 450–457. 10.1097/qai.0000000000001693 29874201

[B3] Athiyyah A. F. Brahmantya H. Dwiastuti S. Darma A. Puspitasari D. Husada D. (2019). Effect of Lactobacillus Plantarum IS-10506 on Blood Lipopolysaccharide Level and Immune Response in HIV-Infected Children. Iran J. Microbiol. 11, 137–144. 31341568 PMC6635306

[B4] Carter G. M. Esmaeili A. Shah H. Indyk D. Johnson M. Andreae M. (2016). Probiotics in Human Immunodeficiency Virus Infection: A Systematic Review and Evidence Synthesis of Benefits and Risks. Open Forum Infect. Dis. 3, ofw164. 10.1093/ofid/ofw164 27747250 PMC5063545

[B5] Ceccarelli G. Statzu M. Santinelli L. Pinacchio C. Bitossi C. Cavallari E. N. (2019). Challenges in the Management of HIV Infection: Update on the Role of Probiotic Supplementation as a Possible Complementary Therapeutic Strategy for cART Treated People Living with HIV/AIDS. Expert Opin. Biol. Ther. 19, 949–965. 10.1080/14712598.2019.1638907 31260331

[B6] Cunninghamrundles S. Ahrne S. Bengmark S. Johann-Liang R. Marshall F. Metakis L.. (2000). Probiotics and Immune Response. Am. J. Gastroenterol. 95, S22–S25. 10.1016/s0002-9270(99)00813-8 10634225

[B7] D'Angelo C. R. M. Costantini E. (2017). Microbiota and Probiotics in Health and HIV Infection. Nutrients 9, 615. 10.3390/nu9060615 PMC5490594 28621726

[B8] d'Ettorre G. Ceccarelli G. Giustini N. Serafino S. Calantone N. De Girolamo G.. (2015). Probiotics Reduce Inflammation in Antiretroviral Treated, HIV-Infected Individuals: Results of the "Probio-HIV" Clinical Trial. PloS one 10, e0137200. 10.1371/journal.pone.0137200 26376436 PMC4573418

[B9] d'Ettorre G. Rossi G. Scagnolari C. Andreotti M. Giustini N. Serafino S. (2017). Probiotic Supplementation Promotes a Reduction in T-Cell Activation, an Increase in Th17 Frequencies, and a Recovery of Intestinal Epithelium Integrity and Mitochondrial Morphology in ART-Treated HIV-1-Positive Patients. Immun. Inflamm. Dis. 5, 244–260. 10.1002/iid3.160 28474815 PMC5569369

[B10] Duprez D. A. Neuhaus J. Kuller L. H. Tracy R. Belloso W. De Wit S. (2012). Inflammation, Coagulation and Cardiovascular Disease in HIV-Infected Individuals. PloS one 7, e44454. 10.1371/journal.pone.0044454 22970224 PMC3438173

[B11] Enos M. K. Burton J. P. Dols J. Buhulata S. Changalucha J. Reid G. (2013). Probiotics and Nutrients for the First 1000 Days of Life in the Developing World. Beneficial Microbes 4, 3–16. 10.3920/bm2012.0020 23257014

[B12] Falasca K. Vecchiet J. Ucciferri C. Di Nicola M. D'Angelo C. Reale M. (2015). Effect of Probiotic Supplement on Cytokine Levels in HIV-Infected Individuals: A Preliminary Study. Nutrients 7, 8335–8347. 10.3390/nu7105396 26426044 PMC4632416

[B13] Gautam N. Dayal R. Agarwal D. Kumar R. Singh T. P. Hussain T. (2014). Role of Multivitamins, Micronutrients and Probiotics Supplementation in Management of HIV Infected Children. Indian J. Pediatr. 81, 1315–1320. 10.1007/s12098-014-1407-6 24760382

[B14] Gismondo M. R. Drago L. Lombardi A. (1999). Review of Probiotics Available to Modify Gastrointestinal flora. Int. J. Antimicrob. Agents 12, 287–292. 10.1016/s0924-8579(99)00050-3 10493604

[B15] González-Hernández L. A. Jave-Suarez L. F. Fafutis-Morris M. Montes-Salcedo K. E. Valle-Gutierrez L. G. Campos-Loza A. E. (2012). Synbiotic Therapy Decreases Microbial Translocation and Inflammation and Improves Immunological Status in HIV-Infected Patients: a Double-Blind Randomized Controlled Pilot Trial. Nutr. J. 11, 90. 10.1186/1475-2891-11-90 23101545 PMC3494555

[B16] Heller F. Duchmann R. (2003). Intestinal flora and Mucosal Immune Responses. Int. J. Med. Microbiol. 293, 77–86. 10.1078/1438-4221-00246 12755368

[B17] Hemsworth J. C. Hekmat S. Reid G. (2012). Micronutrient Supplemented Probiotic Yogurt for HIV-Infected Adults Taking HAART in London, Canada. Gut microbes 3, 414–419. 10.4161/gmic.21248 22825497

[B18] Hummelen R. Changalucha J. Butamanya N. L. Koyama T. E. Cook A. Habbema J. D. F. (2011a). Effect of 25 Weeks Probiotic Supplementation on Immune Function of HIV Patients. Gut Microbes 2, 80–85. 10.4161/gmic.2.2.15787 21637031

[B19] Hummelen R. Hemsworth J. Changalucha J. Butamanya N. L. Hekmat S. Habbema J. D. F. (2011b). Effect of Micronutrient and Probiotic Fortified Yogurt on Immune-Function of Anti-retroviral Therapy Naive HIV Patients. Nutrients 3, 897–909. 10.3390/nu3100897 22254084 PMC3257740

[B20] Irvine S. L. H. R. Hekmat S. Looman C. W. Habbema J. D. Reid G. (2010). Probiotic Yogurt Consumption Is Associated with an Increase of CD4 Count Among People Living with HIV/AIDS. J. Clin. Gastroenterol. 44, 201–205. 10.1097/mcg.0b013e3181d8fba8 20463586

[B21] Irvine S. L. Hummelen R. Hekmat S. (2011). Probiotic Yogurt Consumption May Improve Gastrointestinal Symptoms, Productivity, and Nutritional Intake of People Living with Human Immunodeficiency Virus in Mwanza, Tanzania. Nutr. Res. 31, 875–881. 10.1016/j.nutres.2011.10.005 22153512

[B22] Klatt N. R. Canary L. A. Sun X. Vinton C. L. Funderburg N. T. Morcock D. R. (2013). Probiotic/prebiotic Supplementation of Antiretrovirals Improves Gastrointestinal Immunity in SIV-Infected Macaques. J. Clin. Invest. 123, 903–907. 10.1172/JCI66227 23321668 PMC3561826

[B23] Kuller L. H. Tracy R. Belloso W. De Wit S. Drummond F. Lane H. C. (2008). Inflammatory and Coagulation Biomarkers and Mortality in Patients with HIV Infection. Plos Med. 5, e203. 10.1371/journal.pmed.0050203 18942885 PMC2570418

[B24] Liu J. Williams B. Frank D. Dillon S. M. Wilson C. C. Landay A. L. (2017). Inside Out: HIV, the Gut Microbiome, and the Mucosal Immune System. J. Immunol. 198, 605–614. 10.4049/jimmunol.1601355 28069756

[B25] Lorenc A. Robinson N. (2013). A Review of the Use of Complementary and Alternative Medicine and HIV: Issues for Patient Care. AIDS Patient Care and STDs 27, 503–510. 10.1089/apc.2013.0175 23991688 PMC3760022

[B26] Monachese M. Cunningham-Rundles S. Diaz M. Guerrant R. Hummelen R. Kemperman R. (2011). Probiotics and Prebiotics to Combat Enteric Infections and HIV in the Developing World: a Consensus Report. Gut microbes 2, 198–207. 10.4161/gmic.2.3.16106 21804356

[B27] Pavlova-McCalla E. Trepka M. J. Ramirez G. Niyonsenga T. (2012). Socioeconomic Status and Survival of People with Human Immunodeficiency Virus Infection before and after the Introduction of Highly Active Antiretroviral Therapy: A Systematic Literature Review. J. AIDS Clin. Res. 3, 1000163. 10.4172/2155-6113.1000163 24575328 PMC3933225

[B28] Pinacchio C. Scheri G. C. Statzu M. Santinelli L. Ceccarelli G. Innocenti G. P. (2018). Type I/II Interferon in HIV-1-Infected Patients: Expression in Gut Mucosa and in Peripheral Blood Mononuclear Cells and its Modification upon Probiotic Supplementation. J. Immunol. Res. 2018, 1738676. 10.1155/2018/1738676 30186879 PMC6109550

[B29] Poorolajal J. Hooshmand E. Mahjub H. Esmailnasab N. Jenabi E. (2016). Survival Rate of AIDS Disease and Mortality in HIV-Infected Patients: a Meta-Analysis. Public health 139, 3–12. 10.1016/j.puhe.2016.05.004 27349729

[B30] Roederer M. Staal F. J. Raju P. A. Ela S. W. Herzenberg L. A. Herzenberg L. A. (1990). Cytokine-stimulated Human Immunodeficiency Virus Replication Is Inhibited by N-Acetyl-L-Cysteine. Proc. Natl. Acad. Sci. 87, 4884–4888. 10.1073/pnas.87.12.4884 2112750 PMC54223

[B31] Salminen M. K. Tynkkynen S. Rautelin H. Poussa T. Saxelin M. Ristola M. (2004). The Efficacy and Safety of ProbioticLactobacillus rhamnosusGG on Prolonged, Noninfectious Diarrhea in HIV Patients on Antiretroviral Therapy: A Randomized, Placebo-Controlled, Crossover Study. HIV Clin. Trials 5, 183–191. 10.1310/6f83-n39q-9ppp-lmvv 15472792

[B32] Santos A. S. E. A. C. S. E. Falco M. O. Nery M. W. Turchi M. D. (2015). Effectiveness of Nutritional Treatment and Synbiotic Use on Gastrointestinal Symptoms Reduction in HIV-Infected Patients: Randomized Clinical Trial. Clin. Nutr. 36, 680–685. 10.1016/j.clnu.2016.06.005 27395330

[B33] Schunter M. Chu H. Hayes T. L. McConnell D. Crawford S. S. Luciw P. A.. (2012). Randomized Pilot Trial of a Synbiotic Dietary Supplement in Chronic HIV-1 Infection. BMC Complement. Altern. Med. 12, 84. 10.1186/1472-6882-12-84 22747752 PMC3414771

[B34] Sharma M. Devi M. (2014). Probiotics: a Comprehensive Approach toward Health Foods. Crit. Rev. Food Sci. Nutr. 54, 537–552. 10.1080/10408398.2011.594185 24237003

[B35] Simone C. D. Ciardi A. Grassi A. Gardini S. L. Tzantzoglou S. Trinchieri V. (1992). Effect ofBifidobacterium bifidumandLactobacillus Acidophiluson Gut Mucosa and Peripheral Blood B Lymphocytes. Immunopharmacology and immunotoxicology 14, 331–340. 10.3109/08923979209009228 1597660

[B36] Stiksrud B. Nowak P. Nwosu F. C. Kvale D. Thalme A. Sonnerborg A. (2015). Reduced Levels of D-Dimer and Changes in Gut Microbiota Composition after Probiotic Intervention in HIV-Infected Individuals on Stable ART. J. Acquir Immune Defic Syndr. 70, 329–337. 10.1097/qai.0000000000000784 26258571

[B37] Suttajit M. (2007). Advances in Nutrition Support for Quality of Life in HIV+/AIDS. Asia Pac. J. Clin. Nutr. 16 (Suppl. 1), 318–322. 17392127

[B38] Sutton A. J. Song F. Gilbody S. M. Abrams K. R. (2000). Modelling Publication Bias in Meta-Analysis: a Review. Stat. Methods Med. Res. 9, 421–445. 10.1177/096228020000900503 11191259

[B39] Trois L. Cardoso E. M. Miura E. (2008). Use of Probiotics in HIV-Infected Children: a Randomized Double-Blind Controlled Study. J. Trop. Pediatr. 54, 19–24. 10.1093/tropej/fmm066 17878180

[B40] Villar-García J. Hernández J. J. Güerri-Fernández R. González A. Lerma E. Guelar A. (2015). Effect of Probiotics (Saccharomyces Boulardii) on Microbial Translocation and Inflammation in HIV-Treated Patients. J. Acquir Immune Defic Syndr. 68, 256–263. 10.1097/qai.0000000000000468 25469528

[B41] Vujkovic-Cvijin I. Dunham R. M. Iwai S. Maher M. C. Albright R. G. Broadhurst M. J. (2013). Dysbiosis of the Gut Microbiota Is Associated with HIV Disease Progression and Tryptophan Catabolism. Sci. translational Med. 5, 193ra191. 10.1126/scitranslmed.3006438 PMC4094294 23843452

[B42] Wolf B. W. Wheeler K. B. Ataya D. G. Garleb K. A. (1998). Safety and Tolerance of Lactobacillus Reuteri Supplementation to a Population Infected with the Human Immunodeficiency Virus. Food Chem. Toxicol. 36, 1085–1094. 10.1016/s0278-6915(98)00090-8 9862651

[B43] Yang O. O. Kelesidis T. Cordova R. Khanlou H. (2014). Immunomodulation of Antiretroviral Drug-Suppressed Chronic HIV-1 Infection in an Oral Probiotic Double-Blind Placebo-Controlled Trial. AIDS Res. Hum. Retroviruses 30, 988–995. 10.1089/aid.2014.0181 25127924 PMC6461151

